# Comment on “Role of Mitochondrial Genome Mutations in Pathogenesis of Carotid Atherosclerosis”

**DOI:** 10.1155/2018/4575821

**Published:** 2018-03-28

**Authors:** Josef Finsterer, Sinda Zarrouk-Mahjoub

**Affiliations:** ^1^Krankenanstalt Rudolfstiftung, Vienna, Austria; ^2^University of Tunis El Manar and Genomics Platform, Pasteur Institute of Tunis, Tunis, Tunisia

We read with interest the article by Sazonova et al. about their study of 700 patients with atherosclerosis who were investigated for the presence or absence of 11 mtDNA variants [1]. The authors found that the mtDNA variants m.652delG, m.3336C>T, m.12315G>A, m.14459G>A, and m.15059G>A could serve as biomarkers for the assessment of future atherosclerotic risk in the general population [1]. We have the following comments and concerns.

We do not agree with the statement that the above-mentioned mtDNA variants could be useful as a biomarker of atherosclerosis [1] for several reasons. First, heteroplasmy rates were determined only in blood lymphocytes [1]. It is well known that heteroplasmy rates vary considerably between different tissues and that heteroplasmy rates in lymphocytes do not reflect the mutation load in clinically or instrumentally affected tissues. Were the heteroplasmy rates determined in tissues other than lymphocytes? Heteroplasmy rates from vascular endothelial cells or vascular smooth muscle cells would be of interest, at least in an animal model of atherosclerosis.

Second, it is not mentioned how many of the included patients had arterial hypertension, diabetes, and hyperlipidemia or how many were smoking. Since these classical risk factors for atherosclerosis were not assessed, it cannot be excluded that plaque burden in the carotid arteries is in fact attributable to any of the classical risk factors and has nothing to do with the amount of mtDNA variants.

Third, more specific risk factors for atherosclerosis, such as high-sensitivity C-reactive protein, the telomere length, lipid oxidation products (MDA-modified collagen type IV IgM and IgG antibodies), hyperuricemia, TNF-alpha, and IL-15 polymorphism, were not assessed. They also can have a significant influence on the severity and progression of atherosclerosis.

Fourth, we need to be informed about the medication these patients were regularly taking, since there are a number of compounds which potentially increase the risk of atherosclerosis such as anabolic androgen steroids, testosterone gel, antiretroviral agents, proton pump inhibitors, leptin, or antiepileptic drugs, particularly valproate and carbamazepine [2].

It would be also interesting to know how many of those who carried an mtDNA variant also suffered from a mitochondrial disorder (MID) at inclusion or how many developed a MID during follow-up. It is well appreciated that MIDs manifest not only in the arteries but also in the brain, spinal cord, eyes, ears, endocrine organs, heart, lungs, intestines, kidneys, blood, bones, or skin [3]. In the included patients, were any of these organs or tissues affected alone or in combination? In the included patients, was the family history positive for a MID?

It is also well known that large and small arteries may be affected in MIDs manifesting as dissection of the carotid arteries [4–6], aneurysm formation [5], ectasia of the aorta [7], microangiopathy of the retinal arteries [8], spontaneous rupture of the aorta [9], premature atherosclerosis [10], arteriovenous malformations [11], and arterial hypertension [12]. How many of the included patients manifested with arteriopathy other than atherosclerosis?

Overall, this interesting study could profit not only from more widespread genetic studies but also from the assessment of common and uncommon risk factors for atherosclerosis to exclude influences other than the amount of mtDNA variants tested on the atherosclerotic risk in the general population.
